# Rapid Isolation of Low-Level Carbapenem-Resistant *E. coli* from Water and Foods Using Glycan-Coated Magnetic Nanoparticles

**DOI:** 10.3390/bios13100902

**Published:** 2023-09-23

**Authors:** Oznur Caliskan-Aydogan, Saad Asadullah Sharief, Evangelyn C. Alocilja

**Affiliations:** 1 Department of Biosystems and Agricultural Engineering, Michigan State University, East Lansing, MI 48824, USA; oznurca@msu.edu (O.C.-A.); shariefs@msu.edu (S.A.S.); 2 Global Alliance for Rapid Diagnostics, Michigan State University, East Lansing, MI 48824, USA

**Keywords:** glycan-coated magnetic nanoparticles, carbapenemase-producing *E. coli*, magnetic extraction

## Abstract

Carbapenem-resistant *Enterobacterales* (CRE) are one of the major global issues needing attention. Among them, carbapenemase-producing (CP) *E. coli* strains are commonly found in clinical and biological samples. Rapid and cost-effective detection of such strains is critical in minimizing their deleterious impact. While promising progress is being made in rapid detection platforms, separation and enrichment of bacteria are required to ensure the detection of low bacterial counts. The current separation methods, such as centrifugation, filtration, electrophoresis, and immunomagnetic separation, are often tedious, expensive, or ineffective for clinical and biological samples. Further, the extraction and concentration of antimicrobial-resistant bacteria (ARB) are not well documented. Thus, this study assessed the applicability of cost-effective glycan-coated magnetic nanoparticles (gMNPs) for simple and rapid extraction of CP *E. coli*. The study included two resistant (R)strains: *Klebsiella pneumoniae* carbapenemase (KPC)-producing *E. coli* (R: KPC) and New Delhi metallo-β-lactamase (NDM)-producing *E. coli* (R: NDM). A susceptible *E. coli* (S) strain was used as a control, a reference bacterium. The gMNPs successfully extracted and concentrated *E. coli* (R) and *E. coli* (S) at low concentrations from large volumes of buffer solution, water, and food samples. The gMNPs concentrated up to two and five times their initial concentration for *E. coli* (R) and *E. coli* (S) in the buffer solution, respectively. In water and food samples, the concentration of *E. coli* (S) and *E. coli* (R) were similar and ranged 1–3 times their initial inoculation. A variation in the concentration from different food samples was seen, displaying the impact of food microstructure and natural microflora. The cost-effective and rapid bacterial cell capture by gMNPs was achieved in 15 min, and its successful binding to the bacterial cells in the buffer solution and food matrices was also confirmed using Transmission Electron Microscopy (TEM). These results show promising applications of gMNPs to extract pathogens and ARB from biological samples.

## 1. Introduction

In the growing global phenomenon of infectious diseases, infections by antimicrobial-resistant bacteria (ARB) are a major concern [1]. The emergence and spread of ARB are linked with the overuse and misuse of antibiotics in healthcare, veterinary medicine, and agriculture, their release into the environment [2,3,4,5], and their gene transmission through horizontal gene transfer (HGT) [4,5,6]. Thus, several microorganisms have been identified as a cause of severe common infections due to acquired resistance to one or more antibiotics on the market, which are found in people, animals, food, plants, and the environment [6,7]. To control and minimize the effect of ARB, there have been great research efforts during the last decade for their rapid detection and to make better-informed antibiotic choices.

Infections related to carbapenem-resistant bacteria have been alarmingly increasing in the last decade and are on the global priority list of ARB [5,8,9]. Even though carbapenems are used as the last line of defense in severe infections in humans, several studies showed that carbapenem-resistant *Enterobacterales* (CRE) are also found in food-producing animals, foods, and water sources, among others [5,6,7,10,11,12,13,14]. The emergence and spread of CRE are mainly a result of the rapid dissemination of the carbapenemase-producing (CP) gene through HGT [15]. The most prevalent carbapenemases are *Klebsiella pneumoniae* carbapenemase (KPC) and New Delhi metallo-β-lactamase (NDM) [5,16]. To prevent their emergence and spread in the community, antimicrobial resistance (AMR) monitoring systems routinely test the presence of carbapenemases and the resistant profile of specific pathogens in clinical and biological samples [8,17,18,19].

Rapidly detecting CRE from matrices can assist in preventing or minimizing the associated health and economic consequences [1]. Several phenotypic tests such as matrix-assisted laser desorption/ionization time-of-flight mass spectrometry (MALDI-TOF MS), colorimetric assays (Carba NP tests), amplification-based molecular tests, and biosensors have been implemented to detect CRE in a simple, rapid, and cost-effective manner [7,20,21,22]. The detection limit or sensitivity of these techniques was variable with ≥10^3^ CFU/mL for MALDI-TOF MS, CarbaNP tests, and electrochemical and optical biosensors, while genotypic methods require 10^1^–10^3^ CFU/mL [7,22]. However, these techniques have usually used pure or overnight cultures to ensure enough bacterial count for bacterial detection and for determining their resistant profile [7]. Further, the isolation of ARB, including CRE, from various matrices has not explicitly been documented well. A few examples exist for separating CRE from pure cultures and clinical samples [23,24,25,26], but data are scarce for food matrices. For rapid and sensitive detection of CRE, their direct extraction (isolation) from matrices in a rapid and simple way is therefore of utmost importance and needs attention.

Overall, current bacterial separation techniques in food and clinical matrices include physical methods such as centrifugation and filtration and biochemical methods such as dielectrophoresis and magnetic nanoparticle (MNP)-based techniques [27,28,29]. Bacterial separation by filtration and centrifugation is commonly used and is primarily based on bacterial size and solution density [28,29,30]. While these methods are inexpensive and quick, their effectiveness is sometimes limited and remediated by multiple steps due to large food particles or blood cells and platelets in clinical samples, requiring overnight enrichment [28,30,31,32]. Biochemical methods for bacterial separation utilize naturally occurring biological or chemical interactions between affinity ligands and specific surface substrates on solid support [27,28,30]. For example, dielectrophoresis (DEP)-based techniques mainly utilize non-specific affinity ligands for separating a variety of bacterial cells, with negatively charged bacteria adhering to positively charged residues [28,29,33]. This technique is also employed for differentiating susceptible bacteria utilizing changes in dielectrophoretic behaviors related to antibiotic-induced cell wall inhibition and cell lysis [34]. However, separation of ARB from matrices has not been specifically documented. Although this technique has been used for pathogen separation from food and clinical matrices [28,29,30], complex matrices present problems because of the high conductivity of food particles, limiting their use [28,29,30,33]. In biochemical methods, MNP-based techniques have recently been used for rapid and effective concentration of bacteria from complex samples due to their low cost, simplicity, and unique properties (large surface area/volume ratio and superparamagnetic) [28,35,36,37]. MNPs can be functionalized with recognition moieties such as antibodies, phages, carbohydrates, protein groups, and antibiotics for higher bacterial capture at low concentrations [27,35,38,39].

In MNP-based techniques, antibody-based or immunomagnetic separation (IMS) has commonly been used to separate pathogens and resistant bacteria from food and clinical matrices, and often combined with various detection platforms [24,25,40,41,42,43,44]. The IMS has also been implemented with immune-based assays or sensors for analyzing the proteins, enzymes, and genes of target pathogens [45,46,47]. However, food debris can block the antibody, preventing the separation and concentration of bacteria [28,30]. In addition, antibody production and conjugation with MNPs are time-consuming and expensive, along with low-temperature storage requirements, limiting their use in low-resource settings [28]. In MNP-based methods, bacteriophage-based separation has recently been applied to rapidly separate specific bacteria of interest from complex environments [30,48,49]. For example, phage-based MNPs were used to extract bacteria from water and foods such as lettuce, milk, cheese, and salmon [50,51]. However, the method has limitations with a tedious and lengthy process of coating magnetic particles with phages, undesired damage of target cells, and DNA degradation [27,30,48,52].

Further, MNPs functionalized with biomolecules, such as carbohydrates, proteins, and antibiotics, offer inexpensive, simple, and rapid alternatives to antibody and phage-based MNP separation [27]. For example, vancomycin-coated MNPs can successfully recognize the cell surface of various Gram-positive and Gram-negative bacteria [35,38], including vancomycin-resistant *Enterococci* [53] and carbapenem-resistant *E. coli* and *K. pneumoniae* [26], however, there are concerns about their antibacterial activity [54]. Other MNPs, such as those functionalized with amine groups, have been used to extract bacteria from water, green tea, and grape juice, achieving high capture efficiencies [55], but the functionalization procedure takes several hours. Many carbohydrate surfactants or glycans, such as mannose, galactose, glucose, caprylic acid cysteine, and chitosan, have also been used as MNP coatings [27,28,35,56,57]. In carbohydrate-coated MNPs, glycan (chitosan)-coated MNPs (gMNPs) are promising due to their cost-effective, rapid, and efficient bacterial separation, stability at room temperature, scalable production, and compatibility with many detection techniques [28,57,58,59]. For example, gMNPs successfully extracted and concentrated several bacteria from large-volume food samples, including milk [57,59], thick and complex liquids (beef juice, apple cider, and homogenized eggs) [58], sausage, deli ham, lettuce, spinach, chicken salad, and flour [60]. However, the use of gMNPs for the isolation of ARB, specifically CRE, from real samples requires attention.

As CRE is a global concern, rapid extraction of the causative bacteria from matrices is equally important as their rapid detection. Water sources and global food trade are among the primary routes for the export of CRE and their genes, posing a significant risk to human health [10,61,62]. Therefore, there has been a zero-tolerance policy and international ban on the sale of foods contaminated with CRE in several countries [5]. Thus, this research aimed to assess the use of gMNPs for rapid extraction of CRE from water and food samples.

### Platform Novelty and Applicability

This study aimed to expand the application of gMNPs to rapidly extract ARB, specifically carbapenem-resistant *E. coli*, since CP *E. coli* in CRE is commonly found in water sources and foods [5,14,63,64]. The gMNPs were used to extract three *E. coli* isolates: carbapenem-susceptible *E. coli* (S) as a reference (control) bacterium, KPC-producing resistant *E. coli* (R: KPC), and NDM-producing resistant *E. coli* (R: NDM). The gMNPs–bacteria binding capacity in buffer solutions and the effect of cell surface charge were first assessed. Following this, other factors affecting the MNP binding, such as solution pH and bacterial concentrations, were examined to elucidate their potential impact on bacterial capture and binding. Finally, the gMNPs were used to extract these bacteria from a large volume of food and water samples. To our knowledge, this is the first study on the separation of ABR, including CRE, using gMNPs from water and food matrices.

## 2. Material and Methods

### 2.1. Materials

Tryptic Soy Agar (TSA), Tryptic Soy Broth (TSB), Hydrochloric Acid (ACS reagent, 37%), and Phosphate-Buffered Saline (PBS, pH 7.4) were purchased from Sigma Aldrich. Selective media, CHROMagar for *E. coli* and SUPERCARBA for CP bacteria, were purchased from DRG International (Springfield, NJ, USA). Sodium Hydroxide (NaOH) pellets were obtained from VWR International. Transmission Electron Microscope (TEM) supplies (glutaraldehyde, uranyl acetate stain, and cacodylate buffer) were provided by the Center for Advanced Microscopy (CAM), Michigan State University (MSU). Copper grids for TEM (formvar/carbon-coated 200 mesh copper grids) were obtained from Electron Microscopy Systems (Hatfield, PA, USA). Whirl-Pak bags (92 oz. and 18 oz.) were purchased from VWR International. Racks for magnetic separation were purchased from Spherotech (Lake Forest, IL, USA). All food materials were purchased from a local seller and stored at 4 °C before use.

### 2.2. Bacterial Strains and Culture Conditions

Bacterial strains of susceptible *E. coli* C-3000 (ATCC 15597) and KPC-producing carbapenem (imipenem and ertapenem)-resistant *E. coli* (BAA-2340) were obtained from the American Type Culture Collection (ATCC). A bacterial strain of NDM-producing carbapenem-resistant *E. coli* was obtained from the Michigan Department of Health and Human Services (MDHHS). Stock cultures frozen at –80 °C were refreshed on TSA and TSB and incubated at 37 °C for 24–48 h. Fresh bacterial cultures were grown in 9 mL of TSB for each experiment with an overnight incubation at 37 °C. Then, the fresh cultures were transferred to new 9 mL of TSB for 4–6 h incubation with agitation at 125 rpm before the experiment.

### 2.3. gMNPs Synthesis and Characterization

In-house proprietary glycan (chitosan)-functionalized MNPs were prepared in the Nano-Biosensor Lab, MSU, and synthesized following a previously documented procedure [65]. Briefly, an iron oxide (III) or magnetite (Fe_3_O_4_) core with chitosan shells was used to make the MNPs. Ferric chloride hexahydrate (as a precursor), in a mixture of ethylene glycol (as a reducing agent) and sodium acetate (as porogen), and deacetylated chitosan (5 mg/mL) were used for the MNP synthesis. Chitosan was polymerized to surface-modify the iron oxide nanoparticles. Batches of the glycan-coated MNPs were stored at room temperature for further use. The gMNPs are stable for at least 3 years; new synthesis was not required for everyday analysis. The gMNPs were suspended in sterile deionized water and sonicated before each experiment.

The gMNPs were further characterized by measuring their size using a Zetasizer (Zen3600, Malvern, Worcestershire WR14 1XZ, UK); 100 µL of gMNPs was suspended in 900 µL of sterile deionized water; a total of 2 mL of gMNPs for sample measurement were taken directly. The morphology of gMNPs was also analyzed using a Transmission Electron Microscope (TEM) (JEM-1400 Flash, Jeol, Tokyo, Japan). The prepared solution of gMNPs (5 µL) was directly dropped onto the copper grid and washed with distilled water, followed by air-drying prior to imaging.

### 2.4. Visualization of gMNPs–Bacteria Binding

**Scheme 1:**
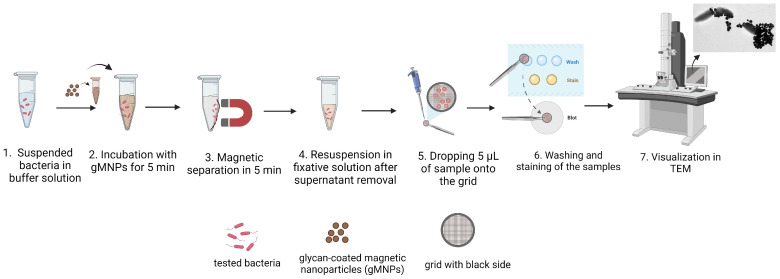
The general procedure of TEM imaging; MNP–bacterial cell interaction (created with BioRender.com, accessed on 4 August 2023).

Visualization of the gMNPs interaction with bacterial cells was assisted by TEM using a standard negative-staining procedure, as illustrated in Scheme 1. For TEM imaging, a few colonies of the overnight culture on TSA were dissolved in 900 µL PBS. After the magnetic incubation and separation, the samples were resuspended in 100 µL fixative solution (2.5% of glutaraldehyde in 0.1 M cacodylate buffer) and followed by staining. The staining procedure used 5 µL of the sample dropped onto the copper grid with a black side for 20–30 s, following which 5 µL of 0.5% uranyl acetate stain was added, and the excess stain was removed. The grids were loaded into the specimen holder of the TEM, and images were taken in the range of 5000–25,000× magnification.

### 2.5. Cell Surface Charge (Zeta Potential) Measurement

The zeta potential of the susceptible and resistant *E. coli* isolates, pure gMNPs, and gMNPs–bacteria conjugation was measured using a Zetasizer. First, the fresh cultures were grown for 4–6 h in TSB, and samples with similar optical density (OD 600) values (~0.5), confirmed using NanoDrop One C, were used. Then, the bacterial culture was centrifuged (Eppendorf AG, 22331 Hamburg, German) at 10,000 rpm for 3 min. After removing the supernatant, the pellet was resuspended in 1 mL of sterile deionized water. For zeta potential measurement of pure gMNPs solution and gMNPs–bacteria conjugation, 100 µL of gMNPs were separately suspended in 900 µL of sterile deionized water and 900 µL of bacteria suspended water, with 5 min incubation. Finally, the resuspended samples were loaded into a folded capillary cuvette and placed into the instrument for measurements. Experiments were performed in triplicates.

### 2.6. Bacterial Extraction in Buffer Solutions

The gMNP-based bacterial extraction or concentration factor (CF) was quantified through plating (colony counting) on solid growth media [28]. First, 4–6 h spiked bacterial cultures were serially diluted in PBS (pH: 7.4) to a concentration of approximately 10^3^ and 10^2^ CFU/mL, plated for initial bacterial counts. For the treatment group, 100 µL of gMNPs was added into 900 µL of the diluted bacterial samples (10^3^ and 10^2^ CFU/mL), vortexed for 10–15 s, and then allowed to stand for 5 min. After 5 min of magnetic separation, the supernatant was removed and resuspended in 100 µL of PBS. Finally, the separated samples were plated on TSA and left for 24 h for incubation at 37 °C. The gMNPs–bacteria binding capacity was determined by CF through colony counts from control samples (no MNP treatment) and MNP-treated samples; only plates with ~20–200 colonies were used. The CF was calculated separately for each serial trial using the following formula (Equation (1)). Experiments were performed in triplicates.

(1) $$Concentration\;Factor=\frac{number\;of\;colony\;in\;gMNPs-treated\;samples}{numebr\;of\;colony\;in\;control\;samples}$$

Following the confirmation of bacterial extraction, the effect of different bacterial concentrations and PBS adjusted to varying pH levels was conducted with the same procedure. For pH analysis, the PBS pH was first adjusted using HCl or NaOH to 3, 4, 5, 6, 7, and 8, confirmed using a pH meter. The procedure was conducted in triplicate at each pH level with a fixed bacterial load (~10^3^ CFU/mL). For the effect of bacterial concentration, fresh 4–6 h spiked bacterial cultures were serially diluted from 10^7^ to 10^2^ CFU/mL, and CF was determined. The bacterial concentration experiments were conducted in PBS with a fixed pH of 7.4. Each experiment was performed in triplicates.

### 2.7. Bacterial Extraction from Large-Volume Samples (Foods and Water)

**Scheme 2:**
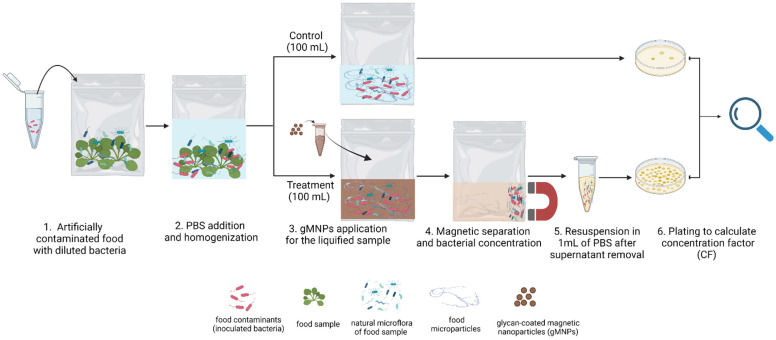
The general procedure of gMNP-based bacterial extraction from large-volume samples (Created with BioRender.com, accessed on 10 June 2023).

Matrices chosen for this study include romaine lettuce, raw chicken breast, ground beef, and tap water. The procedure for magnetic extraction was modified according to the Bacteriological Analytical Manual (BAM) protocols. Scheme 2 illustrates the general method used for the extraction. First, 25 g of each food sample or 25 mL of PBS or water in a Whirl-Pak bag were separately inoculated with 1 mL of ~10^5^ CFU/mL of fresh bacterial culture. Each experiment also had an uninoculated negative control group to account for natural microflora. After 1 h of room temperature acclimation of the contaminated samples, 225 mL of PBS was added and homogenized in a stomacher for 2 min. The liquified food matrix was then separated into two Whirl-Pak bags with 100 mL each, one serving as a no gMNPs (control) and the other as a test. To the test sample, 1 mL of gMNPs were added, mixed, and incubated at room temperature for 5 min. After 5 min magnetic separation and supernatant removal, the extracted cells (attached to the bag) were resuspended in 1 mL PBS. The separated gMNPs–bacteria mixture and control samples were plated on the selective media CHROMagar for *E. coli* identification and SUPERCARBA for CP bacteria identification. Also, uninoculated samples were treated with gMNPs; their control and gMNPs-treated samples were plated on both selective and TSA plates to account for natural microflora and confirm the absence of the interested bacteria. The plates were incubated at 37 °C for 24–48 h. For CF of *E. coli* (S), CP *E. coli* (R), and natural microflora from water and food samples, the number of blue colonies on CHROMagar plates, pink colonies on SUPERCARBA plates, and all colonies on TSA plates were used, respectively. The CF values were calculated based on the Equation (1) as described earlier. Each experiment was performed in triplicates.

Further, the gMNPs–bacteria interaction from the matrices was visualized using TEM. The extracted gMNPs–cells were resuspended in 100 µL fixative solution (2.5% of glutaraldehyde in 0.1 M cacodylate buffer). A 5 µL of the mixture was dropped onto the copper grid and followed by the negative staining as described earlier (Scheme 1).

## 3. Results and Discussion

### 3.1. Characterization of gMNPs

**Figure 1:**
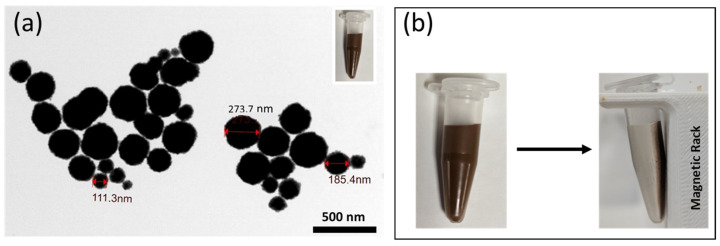
Characterization of the synthesized gMNPs: (**a**) TEM micrograph of the gMNPs with an inset tube for gMNPs solution and (**b**) visualization of superparamagnetic properties of gMNPs under external magnet.

The synthesized gMNPs were characterized using TEM and Zetasizer. The suspension of gMNPs in sterile deionized water and its electron micrograph are illustrated in Figure 1a. All particles were roughly spherical, with multiple gMNPs of varying size, and clumping of particles was usually observed. The size (diameter) of gMNPs in the micrograph was found to be in a range of approximately 40–300 nm, using a measurement tool in TEM software. The mean particle size measured by the Zetasizer was found to be 286.5 ± 5.7 nm. The variation in size obtained from TEM and Zetasizer could probably have resulted from the random clumping of the nanoparticles and surface coating of iron oxide nanoparticles. The nanoparticles’ size is critical for their superparamagnetic properties; iron oxide nanoparticles with 50–180 nm showed the highest superparamagnetic properties in an earlier study [66].

Superparamagnetic properties of gMNPs were further confirmed by subjecting the solution to an external magnet for one minute. As seen in Figure 1b, the particles were all separated from the solvent and pulled out to the side of the tube, resulting in clear water. The superparamagnetic properties of gMNPs were also confirmed in an earlier study; the gMNPs were observed to be strongly magnetized under an external magnet, further confirmed with their magnetization curves [57]. In addition, the smaller size of MNPs has a higher surface area-to-volume ratio, which can assist them in moving faster than larger particles. Multiple particles penetrate matrix interstices and interact with bacterial cells, improving their bacterial capture [27,28]. In previous studies, the gMNPs successfully extracted several bacteria from various food matrices [28,57,60,67].

### 3.2. Bacterial Extraction from Buffer Solution

#### 3.2.1. Concentration Factor (CF)

**Figure 2:**
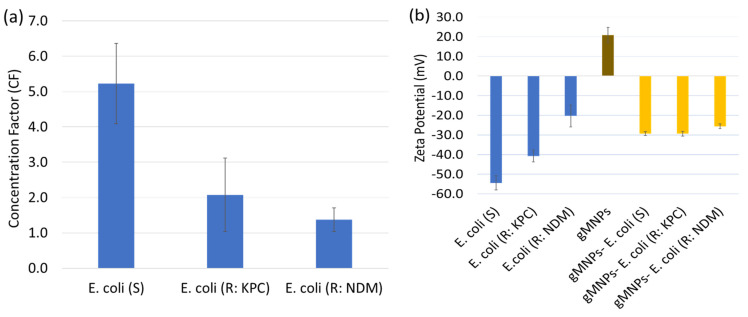
(**a**) The average MNP–bacteria concentration factor, with standard deviations (N: 18) and (**b**) zeta potential for pure bacteria isolates (blue bars);carbapenem-susceptible *E. coli* (S) and carbapenem-resistant *E. coli* (KPC: R) and *E. coli* (R: NDM), pure gMNPs (brown bar), and gMNPs-bacteria conjugation (yellow bars), with standard deviations (N: 9).

The efficacy with which gMNPs extract *E. coli* (S), *E. coli* (R: KPC), and *E. coli* (R: NDM) was investigated using CF. Initially, the CF of these bacteria was determined in a small volume of PBS (1 mL) using the standard plating method; results are illustrated in Figure 2a. The average CF of resistant *E. coli* (R: KPC) and *E. coli* (R: NDM) were ~2 and ~1.4, lower than that for susceptible *E. coli* (S), which displayed a CF of ~5.2. Previous studies have observed differences in bacterial extraction between different bacteria types, which hypothesized electrostatic interaction between positively charged MNPs and negatively charged bacterial cell walls [28,35,68,69]. The chemical nature of bacterial cells and the hydrophilic and hydrophobic groups on cell walls results in differences in the cell surface charge [68].

The zeta potential of the pure bacterial isolates, pure gMNPs, and gMNPs–bacteria conjugations was measured further to understand the effect of surface charge on CF. As seen in Figure 2b, the average zeta potential of *E. coli* (S) was found to be around −54 mV, while *E. coli* (R: KPC) and *E. coli* (R: NDM) were −41 mV and −20 mV, respectively. The zeta potential of resistant *E. coli* (R) isolates was less than that of *E. coli* (S). An earlier study observed similar results; colistin-susceptible *Acinetobacter baumannii* cells had a higher zeta potential compared to that of colistin-resistant *Acinetobacter baumannii* [70].

The average zeta potential of gMNPs was positively charged and found to be around 20 mV. Further, the average zeta potential of gMNPs–bacteria conjugate was approximately −29 mV for *E. coli* (S) and *E. coli* (R: KPC) and −25 mV for *E. coli* (R: NDM).

The positively charged nature of nanoparticles has widely been used in previous studies for the capture of bacterial cells. For example, positively charged gold nanoparticles showed a greater attachment ability to Gram-positive bacteria with higher negative charge than Gram-negative bacteria with lower negative charge [69]. In another recent work, the extraction of Gram-negative *E. coli* O157 was lower (CF = ~4.5) compared to that of Gram-positive *L. monocytogenes* (CF = ~31.6) and *S. aureus* (CF = ~61.2) [60]. Confirming earlier reports, the average zeta potential of *E.coli* O157 is less negative (~−4 mV) than that of *L. monocytogenes* (~−35 mV) and *S. aureus* (~−42 mV) [60]. Similar results were seen in another study, with *S. aureus*, *A. baumanni*, and *P. aeruginosa* displaying a zeta potential of −50.4 mV, −23.9 mV, and −34.6, respectively. Their capture efficiency using polyethyleneimine-modified magnetic microspheres (Fe_3_O_4_@PEI) was found to be 97.87% for *S. aureus*, 80.57% for *A. baumannii*, and 97.25 for *P. aeruginosa* [71]. The chemical nature of cells or environmental conditions leads to differences in cell wall composition, changes in anionic and cationic species on the cell surface, and electrical potential differences [68,69,72,73,74]. In accordance with earlier reports, our study confirmed that the MNP-based bacterial concentration is inversely related to the zeta potential.

The MNP-based bacterial extraction does not only depend on the electrostatic interaction but it is also related to receptor–ligand interactions, which may occur on some portions of the bacterial adhesin surface. Bacterial adherence to surfaces is facilitated by adhesins (polypeptides (fimbrial (pili) or afimbrial) or polysaccharides (usually components of the bacterial cell membrane, cell wall, and capsule)) [75,76]. Each bacteria type has unique components and adhesion mechanisms, regardless of susceptibility status [76,77,78]. Further, several studies highlighted alterations in the biosynthesis of cell wall material, membrane components, and cytoplasmic contents in ARB, resulting in changes in cell surface characteristics and their adhesion or attachment to host surfaces [42,68,70,74,79,80]. For example, an alteration in the lipid A structure of cell walls reduces its negative charge, resulting in the electrostatic repulsion of cationic peptides to the cell wall [81]. Thus, the lower concentration factor of resistant *E. coli* (R) isolates compared to *E. coli* (S) may be additionally related to alteration or distortion in receptor–ligand interaction. Microscopy was used to elucidate the MNP–bacteria binding interactions further.

#### 3.2.2. Microscope Imaging of gMNPs–Bacteria Interaction

**Figure 3:**
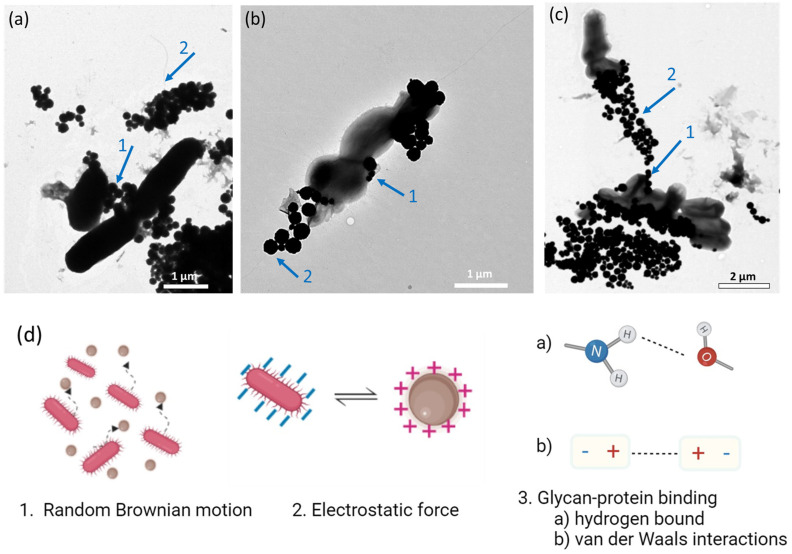
TEM images of MNP interactions with the susceptible and resistant *E. coli* isolates extracted from buffer solution: (**a**) *E. coli* (S), (**b**) *E. coli* (R: KPC), and (**c**) *E. coli* (R: NDM). Arrows show MNP–bacteria interaction: MNP–cell (1) and MNP–flagellum (2). (**d**) Schematic representation of the underlying hypothesis of the gMNPs interaction (created with BioRender.com, accessed on 7 August 2023)), which was adapted by a study [28].

The gMNPs–bacteria interaction was characterized using TEM to confirm the binding. Figure 3 shows roughly spherical gMNPs successfully attached to bacterial cells. Multiple gMNPs on individual and bacterial cell clusters may have contributed to improved bacterial extraction. Their interaction with flagella was also clearly seen in *E. coli* (S) and *E. coli* (R). Earlier studies showed similar MNP–bacterial cell interaction through microscopic imaging [28,57,82]. Multiple gMNPs interacting with individual cells of *E. coli* O157, and clusters of *S. aureus* and *L. monocytogenes* were seen [60]. The interaction was hypothesized to be related to the differences in cell morphology and cell wall components in each bacteria type. Further, the morphological characteristics of bacteria can impact their Brownian forces and cell attachment to surfaces [28,60,83].

The images also illustrated that gMNPs did not cover the entire surface of bacteria, binding only to some portions of the bacterial surface. This might be the effect of location-specific glycan–protein interaction., Tthe established phenomenon of surface glycan attaching to proteins (e.g., lectins) on the bacterial cell surface may help better understand gMNP binding [27,28,35,67,84]. For example, the adhesin FimH can recognize and bind to terminal mannose residues [85], and sugar moieties can bind to concanavalin A protein [86] and C-type Salmo Salar Lectin (SSL) [87].

As earlier studies highlighted, the binding of gMNPs to bacterial cells is usually achieved through a combination of forces (Figure 3d): (1) Brownian motion, which allows random and uncontrolled movement of particles, (2) electrostatic forces, which assist in bringing the positively charged MNPs closer to negatively charged bacterial cells, and (3) in proximity, bacteria can adhere to glycan surface of MNPs through (glycan–protein) hydrogen bonding and van der Waals interaction [28,60,67]. This study showed that these interactions have successfully allowed gMNPs to capture resistant *E. coli* (R) isolates.

The gMNPs interaction with resistant *E. coli* isolates (R: KPC and R: NDM) was seen as clusters, unlike individual cells of *E. coli* (S). Apart from the differences in gMNPs–cell binding, the *E. coli* (R) cells appear smaller and as imperfect rods (bacilli). As previously mentioned, the differences in cell morphology may have resulted from alterations in the cell surface components. In various studies, morphological and ultrastructural changes in bacterial cells under antibiotic exposure were observed and attributed to cell wall synthesis disruption due to deficiency in cell wall materials and cell lysis [68,80,88,89,90,91,92], resulting in a change of cell morphology [80,83] and leading to alterations in electrostatic surface charge [68,70,93,94,95]. For example, carbapenems enter the bacteria through outer membrane proteins (porins) and degrade the penicillin-binding proteins (PBPs) at the cell wall, weakening the glycan backbone in the cell wall and resulting in alteration in cell morphology (filament or non-perfect round shapes) and surface components [20,96,97]. Thus, the distortion or alteration in cell components, including surface proteins, may have affected the glycan–protein interaction for carbapenem-resistant bacteria. 

Considering the antimicrobial properties of chitosan-coated MNPs on bacteria, a previous study has shown that their antibacterial activity and cell lysis of bacteria occur after exposure of at least 8 h [60,98]. Therefore, the short-term (<15 min) exposure of the MNPs to bacteria may not have impacted their cell viability and properties. CP *E. coli* (R) cells before gMNP exposure also showed similar cell morphology (data not shown). Overall, it should be noted that the morphological nature of the resistant bacteria could have impacted their receptor–ligand (glycan–protein) interaction and the electrostatic repulsion between bacteria and gMNPs.

This study showed that the gMNPs could successfully isolate resistant *E. coli* (R) isolates. The bacterial extraction or isolation from the large-volume samples with varying pH environments and bacterial loads was tested to determine their possible field application.

#### 3.2.3. Effect of Bacterial Load and Buffer pH on Concentration Factor

**Figure 4:**
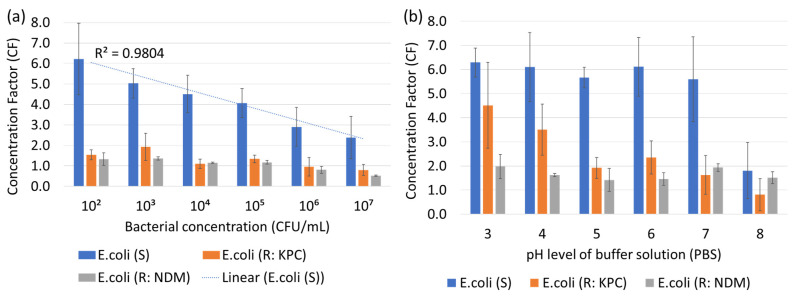
MNP–bacteria binding capacity: (**a**) Average concentration factor at varying bacterial concentrations with standard deviations (N: 9) and (**b**) pH levels of buffer solutions with standard deviations (N: 9).

The extraction of *E. coli* (S) and resistant *E. coli* (R: KPC and R: NDM) was further investigated at varying bacterial concentrations and pH levels to elucidate the binding capacity of MNP (Figure 4). With varying bacterial concentrations at an approximately neutral pH (7.4) of PBS, the average CF of *E. coli* (S) was higher (>~5) at 10^2^–10^3^ CFU/mL, following a linearly decreasing trend as bacterial concentration increased (R^2^ = 0.98) as seen in Figure 4a. However, resistant *E. coli* (R: KPC and R: NDM) isolates did not show a strong linear trend with varying bacterial concentrations (R^2^ = 0.68 and 0.85, respectively). The average CF of *E. coli* (R) isolates was more than 1 at 10^2^–10^5^ CFU/mL, while a lower CF (<1) was seen at 10^6^–10^7^ CFU/mL. Notably, *E. coli* (S) and CP *E. coli* (R) isolates displayed the highest CF at low concentrations (10^2^–10^3^ CFU/mL). Similar results were previously seen in another study where positively charged MNPs successfully captured *E. coli* (90%) at ultra-low concentrations (10–100 CFU/mL) [23]. It was also found that positively charged nanoparticles have a higher adsorption capacity for bacilli (*E. coli* and *B. subtilis*) than staphylococci (*S. aureus*) and streptococci (*L. lactis*) [23]. The higher bacterial extraction with gMNPs at lower bacterial concentrations could assist their quick detection for preventing outbreaks.

The concentration factor was also tested in different pH environments for *E. coli* (S) and resistant *E. coli* (R) isolates (at ~10^3^ CFU/mL). As observed in Figure 4b, *E*. *coli* (S) displayed similar CF (>5) at pH 3–7, while a sharp decrease in CF (~2) at pH 8 was seen. *E. coli* (R: KPC) showed higher CF (~>3) at pH 3–4 level, and the trend became stable (CF = ~2) at pH 5–7, and showed a sharp decrease (CF: <1) at pH 8. However, a similar trend was not observed in *E. coli* (R: NDM); their CF was between 1–2. Overall, these results confirmed earlier reports that reducing environmental pH might impact bacterial extraction. In a previous study, for instance, MNPs in the pH range of 5–8 displayed higher bacterial capture [23,57]. Further, the capture capacity at varying pH levels may also depend on the bacteria type. For example, an earlier report showed that the CF for *E. coli* O157 decreased linearly as the pH level (5–8) increased and remained stable at ~pH 8–10. The same study observed that the CF for *L. monocytogenes* and *S. aureus* did not show a trend and were similar in the pH 6–9 range [60]. Studies have also shown lower pH is generally associated with higher capture [23,57,60]. Since the gMNPs are naturally hydrophilic, the amino groups can be protonated at low pH levels, increasing the charge difference and promoting MNP–bacteria binding capacity [23,57,60].

### 3.3. Bacterial Extraction at Low Concentration from Large-Volume Samples (100 ML)

**Figure 5:**
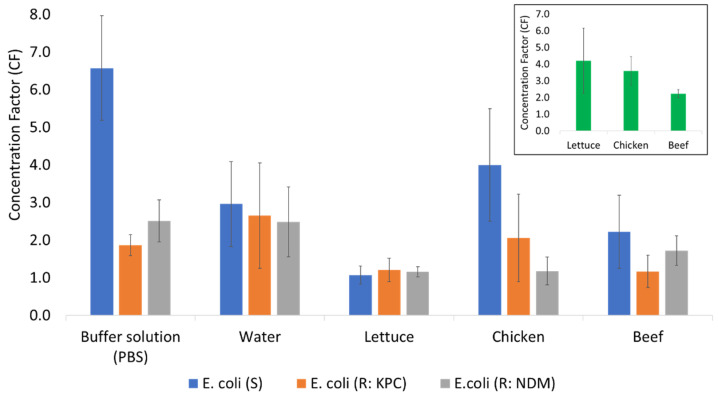
The bacterial extraction from food samples: average concentration factor (CF) from large-volume samples, along with inset figure for CF of uninoculated food samples (control)*,* with standard deviations (N: 18).

To evaluate the applicability of the gMNPs for large-volume bacterial extraction at low concentrations (~10^3^ CFU/mL), the susceptible and resistant *E. coli* isolates were first tested in 100 mL of PBS. The gMNPs successfully extracted *E. coli* isolates, with an average CF of 6.5 for *E. coli* (S), 1.8 for *E. coli* (R: KPC), and 2.5 for *E. coli* (R: NDM); results are shown in Figure 5. Following the large-volume PBS concentration, the applicability of the gMNPs in water and food samples was tested. Magnetic extraction of the bacteria from water and various food types, including lettuce, raw chicken meat, and ground beef, was quantified using CF by selective plating. As seen in Figure 5, *E. coli* (S), *E. coli* (R: KPC), and *E. coli* (R: NDM) were successfully extracted from water and the food matrices with a CF > 1. Although *E. coli* (S) showed higher CF in PBS, the CF was lower in the water and food samples. The bacterial extraction was lower in the lettuce sample compared to chicken, beef, and water samples, which could have been due to the high level of natural microflora in the lettuce samples.

The natural microflora from uninoculated food samples (control) was also magnetically extracted, and their capture was quantified by plating on TSA. The CF of natural microflora was variable, as seen in the inset of Figure 5. The average CF was the highest in lettuce, followed by chicken and beef samples. Notably, the CF of *E. coli* samples was inversely related to the presence of bacterial loads in the lettuce sample. Although the natural microflora was higher in chicken, the average CF of *E. coli* (S) and *E. coli* (R: KPC) was lower in the beef samples. This could also be related to the physical microstructure and chemical constituents of the food sample. For example, lettuce is rich in cellulose components, while chicken and beef contain higher protein and fat amounts [75]. While the food composition of these tested food samples varies considerably, the food microparticles could also have affected the MNP–bacteria binding. The attachment or binding can differ based on the bacteria type, food matrix, and hydrophobic or hydrophilic surface [75]. An earlier study also stated that negatively charged molecules contaminating plant or animal tissues might interfere with the MNP–bacteria interaction [23,75].

**Figure 6:**
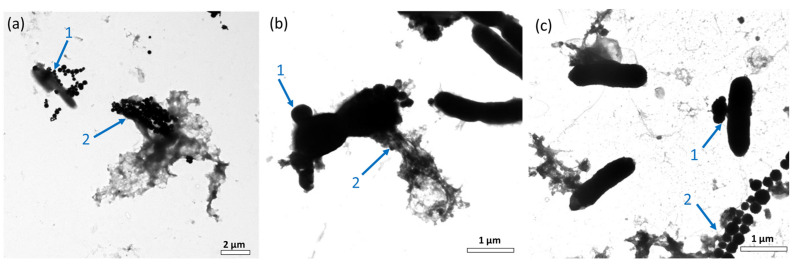
TEM images of MNP–bacteria interactions from inoculated food samples: (**a**) lettuce, (**b**) chicken, and (**c**) ground beef. Arrows show MNP–bacteria interaction: MNP–cell (1) and MNP–microparticles (2).

TEM images were also taken to confirm the binding of the gMNPs to bacterial cells in foods and are shown in Figure 6. The interaction between gMNP and bacterial cells in the presence of food microparticles was confirmed from the micrographs. Images showed that the microparticles (e.g., fat globules, protein fibers, and cellulose) can bind to gMNP and bacteria. In this MNP-based extraction technique, in theory, gMNP–bacteria, bacteria–food matrix, and MNP–food matrix interactions are possible in the liquified sample due to their attachment preferences and electrostatic forces. However, most of the food matrix in the liquefied samples is removed with the supernatant [28]. Magnetic separation using gMNP assists in reducing the remnant food debris from the sample and does not significantly affect the results. Earlier, for example, gMNPs successfully extracted and concentrated several bacteria from large-volume food samples, including milk [57,59], thick and complex liquid (beef juice, apple cider, and homogenized eggs) [58], sausage, deli ham, lettuce, spinach, chicken salad, and flour [60]. Following bacterial extraction from foods using gMNPs, target bacteria were detected, confirming that interferences of the food matrix did not significantly prevent the detection of target DNA [99,100,101]. Thus, the gMNPs are promising as they offer cost-effective, rapid, and efficient bacterial separation from various food matrices [57,58,60].

A detection platform can allow identification of the target bacteria following magnetic extraction, as summarized in Table 1. For example, the magnetic extraction of bacteria from various food matrices was followed by short enrichment, DNA extraction, and plasmonic biosensor detection: *E. coli* from lettuce and spinach [99], *Salmonella* from melons and cucumbers [100], and *E.coli* 0157 from flour [101]. In another work, the glycan/cysteine-coated MNPs captured *E. coli* O157:H7 from vitamin D milk, and then an electrochemical biosensor was used for detection [67].

Magnetic extraction and detection platforms have been used in clinical samples besides bacterial extraction from food samples. For instance, polyethyleneimine-modified magnetic microspheres (Fe_3_O_4_@PEI) were used to capture and enrich bacteria (*S. aureus*, *A. baumannii*, and *P. aeruginosa*) from blood samples by culturing on plates. A single colony from the overnight culture was then used in SERS platforms to identify bacterial type and resistance profile [71]. Elsewhere, vancomycin-modified Fe_3_O_3_-Au NP captured CRE from urine and detected them using carbapenemase hydrolysis activity by a pH meter [26]. In another example, the antibody-conjugated MNP were used to capture methicillin-resistant *S. aureus* (MRSA) from nasal swab at (10^3^–10^5^ CFU/mL), and then an electrochemical sensor was used for their detection [25]. As several studies showed that CRE has been found not only in clinical samples but also in foods and water sources [7,62,102,103,104,105,106,107,108], their rapid extraction directly from water and food samples has not been documented. This work is the first study for the extraction of CRE, specifically CP *E. coli*, from buffer solution and water and food samples. The differences in gMNP-cell binding capacity of resistant *E. coli* (R) compared to *E. coli* (S) may be due to the differences in cell surface characteristics (e.g., morphology and surface charge) of the resistant bacteria. This study has offered further insight into the future potential of magnetic extraction of ARB and their extraction from different matrices.

This study showed the applicability of gMNPs for extracting resistant *E. coli* (R) in food samples in the presence of natural microflora and food microparticles. The bacterial capture in these samples was achieved at concentrations as low as 10^3^ CFU/mL. Notably, many biochemical separation techniques mostly require a concentration above 10^3^ CFU/mL [7]. In addition, this magnetic separation allows accessible and rapid extraction, especially in low-resource settings. The gMNPs can be easily prepared and are chemically stable for three years at room temperature and are cost-effective. For instance, the cost of gMNPs-based extraction was estimated to be as low as USD 0.50 per assay [82,99], compared to IMS, which is USD 5–10 per assay and requires special storage conditions [109]. Further, bacterial extraction and concentration using gMNPs can be achieved within 15 min, while IMS needs longer incubation and extraction time [28].

The non-specific nature of gMNPs may lead to complications in extraction from complex food matrices and those with a high level of natural microflora. The food matrix, in some cases, may affect subsequent detection based on the method employed. In this case, additional washing steps with PBS following supernatant removal can assist in the removal of food particles [28,35,57,84]. However, it should be noted that the non-selective gMNPs allow rapid capturing of several bacterial types and resistant bacteria and do not require specific affinity binding or specific preparation for each bacterial isolation experiment. The non-selective extraction offers to detect more than one possible target bacteria at once. Thus, their implementation of surveillance programs can be helpful to prevent and control the spread of causative bacteria.

**Table 1:** Summary of several MNP-based extractions of *E. coli* and ARB.

MNP Coating	Bacteria	Matrix	Capture	Detection Method	References
PEI-modified gold-coated microspheres	*E. coli*	Tap water Milk	65%	Raman Spectroscopy	[110]
PEI	*E. coli*	Buffer	90%	Plating	[23]
Biotinylated oligosaccharides	*E. coli* (UPEC)	Buffer	17–34%	Luciferase assay	[111]
Antibody	*E. coli* 0157: H7	Whole milk	20%	Colorimetric biosensor	[112]
Antibody	STEC	Apple juice	39–105%	Multiplex qPCR	[113]
Cysteine-glycan	*E. coli* 0157: H7	Vitamin D Milk	73–90%	Plating	[57]
Cysteine-glycan	*E. coli* 0157: H7	Homogenized Egg Milk Apple cider	>70%	Electrochemical biosensor	[58]
Lyseine-SCGs	*E. coli* 0157: H7	Sausage	90%	Colorimetric biosensor	[114]
Antibody	STEC	Ground beef blueberries	NA	Colorimetric biosensor	[115]
Glycan	*E. coli* O157	Flour	NA	Colorimetric biosensor	[101]
Glycan	*E. coli*	Lettuce Spinach	NA	Colorimetric biosensor	[99]
Glycan	*E. coli* O157	Lettuce Spinach Chicken salad Flour	CF: 0.64–2.54	Plating	[60] *
Lectin-silver	*E. coli* MRSA	Buffer	NA	SERS	[23,24]
Antibody	Carbapenem-resistant *A. baumanni*	Culture Sputum	NA	Lateral flow biosensor	[116]
Vancomycin	CRE: *E. coli* and *K. pneumoniae*	Culture Urine	>70%	pH meter sensing	[26]
Antibody	MRSA	Nasal swab	>90%	Electrochemical biosensor	[25]
Glycan	Carbapenem-resistant *E.coli* (KPC and NDM producing)	Lettuce Chicken breast Ground beef Water	CF: 0.94–4.2	Plating (confirming extraction)	This study *

* The specific rapid detection method is not applied. The plating method is for the confirmation of the bacterial extraction. PEI: polyethylemine, STEC: shiga toxin-producing *E. coli*; SCGs: short-chain glucan; MRSA: methicillin-resistant *S. aureus*; CRE: carbapenem-resistant *Enterobacterales*: NA: not applicable.

## 4. Conclusions

Rapid and cost-effective platforms for bacterial extraction from food samples with higher binding efficiency are urgently needed to improve their rapid detection. This study tested glycan-coated MNPs to extract carbapenem-resistant *E. coli* from a buffer solution and large-volume food and water samples. The gMNPs–bacteria binding at varying bacterial concentrations and pH levels was also evaluated. Bacterial extraction from complex food matrices was achieved in the presence of natural microflora and food microparticles and confirmed with microscopic images. This study also showed the potential applicability of gMNPs to extract ARB from various complex solid food matrices. In future work, the applicability and accessibility of this platform can be further tested for the extraction of other ABRs, such as colistin, ampicillin, ESBL, and CRE, and from clinical, environmental, and food samples.

## Data Availability

The data presented in this study are available upon request from the corresponding author.
